# Intracellular distribution of amyloid beta peptide and its relationship to the lysosomal system

**DOI:** 10.1186/2047-9158-1-19

**Published:** 2012-09-26

**Authors:** Lin Zheng, Angel Cedazo-Minguez, Martin Hallbeck, Fredrik Jerhammar, Jan Marcusson, Alexei Terman

**Affiliations:** 1 Division of Geriatric Medicine, Department of Clinical and Experimental Medicine, IKE, Faculty of Health Sciences, Linköping University, Linköping SE-581 85, Sweden; 2 KI-AlzheimerDisease Research Center, NVS, Novum, Karolinska Institutet, Stockholm SE-141 57, Sweden; 3 Division of Pathology, Department of Clinical and Experimental Medicine, IKE, Faculty of Health Science, Linköping University, Linköping SE-581 85, Sweden; 4 Departmentof Pathology, Linköping University Hospital, Linköping SE-581 85, Sweden; 5 Experimental Pathology, Department of Clinical and Experimental Medicine, IKE, Faculty of Health Science, Linköping University, Linkoping SE-581 85, Sweden; 6 Department of Clinical Pathology and Cytology, Karolinska University Hospital, Stockholm SE-141 86, Huddinge, Sweden

**Keywords:** Alzheimer disease, Amyloid β-protein, Colocalization, Exocytosis, Immunocytochemistry, Lysosomes

## Abstract

**Background:**

Amyloid beta peptide (Aβ) is the main component of extraneuronal senile plaques typical of Alzheimer’s disease (AD) brains. Although Aβ is produced by normal neurons, it is shown to accumulate in large amounts within neuronal lysosomes in AD. We have recently shown that under normal conditions the majority of Aβ is localized extralysosomally, while oxidative stress significantly increases intralysosomal Aβ content through activation of macroautophagy. It is also suggested that impaired Aβ secretion and resulting intraneuronal increase of Aβ can contribute to AD pathology. However, it is not clear how Aβ is distributed inside normal neurons, and how this distribution is effected when Aβ secretion is inhibited.

**Methods:**

Using retinoic acid differentiated neuroblastoma cells and neonatal rat cortical neurons, we studied intracellular distribution of Aβ by double immunofluorescence microscopy for Aβ_40_ or Aβ_42_ and different organelle markers. In addition, we analysed the effect of tetanus toxin-induced exocytosis inhibition on the intracellular distribution of Aβ.

**Results:**

Under normal conditions, Aβ was found in the small cytoplasmic granules in both neurites and perikarya. Only minor portion of Aβ was colocalized with trans-Golgi network, Golgi-derived vesicles, early and late endosomes, lysosomes, and synaptic vesicles, while the majority of Aβ granules were not colocalized with any of these structures. Furthermore, treatment of cells with tetanus toxin significantly increased the amount of intracellular Aβ in both perikarya and neurites. Finally, we found that tetanus toxin increased the levels of intralysosomal Aβ although the majority of Aβ still remained extralysosomally.

**Conclusion:**

Our results indicate that most Aβ is not localized to Golgi-related structures, endosomes, lysosomes secretory vesicles or other organelles, while the suppression of Aβ secretion increases intracellular intra- and extralysosomal Aβ.

## Introduction

The mechanisms behind Alzheimer disease (AD), the main cause of senile dementia, are poorly understood. One of the important hallmarks of AD is the formation of extracellular senile plaques, preferentially composed of amyloid beta-protein [1]. The most common isoforms of Aβ are Aβ_40_ (90%) and Aβ_42_ (10%), the latter being more toxic, more prone to aggregation, more resistant to degradation, and specifically increases in all forms of familial AD [2].

Aβ is proteolytically cleaved from a large transmembrane amyloid precursor protein (APP) by β and γ secretases [3]. APP is normally synthesized in the endoplasmic reticulum (ER) and transported to the Golgi apparatus. Eventually it can be trafficked from the trans-Golgi network (TGN) to the cell surface and secreted into extracellular space [4], recycled back to the Golgi complex for further packaging and trafficking [5] or reinternalized from the cell surface into the endosomal-lysosomal system via endocytosis [6-8]. Aβ generation from APP is thought to occur in a variety of organelles where APP, β and γ secretase reside. Thus, Aβ has been found in many intracellular sites, such as ER, Golgi complexes, mitochondria, endosomes, lysosomes, multivesicular bodies (MVB), and cytosol (reviewed in [9]). Autophagic vacuoles have also been shown involved in the production of Aβ [10].

The toxicity of Aβ and its involvement in senile plaque formation are considered important pathophysiological targets for primary prevention in AD (reviewed in [11]). It has been proposed that senile plaques originate from intraneuronal Aβ as a result of its release after neuronal death [12]. Intracellular Aβ has been pointed out to be involved in early stages of the disease, directly causing neurotoxicity and initiating AD pathology [12-19]. It has been reported recently that Aβ –related synapse damage and memory impairment in AD-transgenic mice correlated with intracellular levels of Aβ but not with plaque burden [20]. Moreover, cultured neurons from AD-transgenic mice showed reduced secretion and enhanced intracellular accumulation of Aβ [21]. Much evidence supports that the lysosomal system, a vacuolar compartment with acidic pH (3.5-6.0), is associated with Aβ generation and neurotoxicity [22-26]. In AD and experimental AD models, Aβ has been detected in abnormally enlarged endosomes [12,17,27], autophagosomes [10], and lysosomes [28-30].

Our previous studies showed that normobaric hyperoxia (a chronic, mild oxidative stress) enhanced macroautophagy, inducing intralysosomal Aβ accumulation, lysosomal membrane permeabilization and consequent apoptosis [29-32]. However, it is not clear how Aβ is distributed in relation to the lysosomal system and other organelles normally and how and why this distribution is changed in AD. Here we studied the relation of Aβ to the lysosomal vacuolar compartment (early and late endosomes, lysosomes and autolysosomes) as well as to cellular structures associated with related process of protein secretion (such as Golgi-derived secretory vesicles and synaptic vesicles) using double immunofluorescence microscopy (for Aβ and different organelle markers). RA-differentiated neuroblastoma cells and neonatal rat cortical neurons were used as in vitro models. Cells were cultured under normal conditions as well as in the presence of the exocytosis inhibitor, tetanus toxin (TeNT).

## Materials and methods

### Human neuroblastoma SH-SY5Y cell culture

Human SH-SY5Y neuroblastomacells were obtained from the American Type Culture Collection (Rockville, MD, USA) and cultured in Dulbecco’s Modified Eagle Medium (DMEM; Gibco, Paisley, UK) supplemented with 4500 mg/l glucose, 110 mg/l sodium pyruvate, 584 mg/l glutamine, 10% fetal bovine serum, 50 IU/ml penicillin G and 50 mg/ml streptomycin in 25 cm^2^ plastic culture flasks (Corning, Corning, NY, USA) at 37°C, with 5% CO_2_. For differentiation, neuroblastoma cells were exposed to 10 μM all-trans retinoic acid (RA, Sigma, St. Louis, MO, USA) for 14 days. The medium was changed every second day.

### Neonatal rat cortical neuron culture

Primary culture of neonatal rat cortical neurons was prepared as described previously [33]. Neurons were obtained from the cerebral cortex of newborn Wistar rats and plated onto 35 mm Petri dishes coated with poly-d-lysine (Sigma). The culture medium consisted of DMEM (Gibco) containing 20% fetal bovine serum, 2.5 μg/ml insulin and 45 mM glucose. The percentage of fetal bovine serum was gradually reduced to 5%. The medium was changed twice a week.

### Inhibition of exocytosis

Tetanus toxin (TeNT, Sigma), an exocytosis inhibitor, was used to block the transport of secretory vesicles to the plasma membrane [34]. RA differentiated neuroblastoma cells and primary neurons were treated with 5 or 20 nM tetanus toxin (TeNT) respectively for 24 h.

### Antibodies

Primary anti-Aβ_1–42_ antibodies [35] (Chemicon, Temecula, CA, USA), and anti-Aβ_1–40_ antibodies [36,37] (Chemicon, Temecula, CA, USA), were rabbit polyclonal, while anti-human-Rab8 [38] (marker for TGN and Golgi-derived secretory vesicles, BD biosciences, Franklin Lakes, NJ, USA), anti-Rab9 [39] (marker for TGN and late endosomes, Abcam, Cambridge, UK), anti-Rab5 (marker for early endosomes, Pharmingen, San Diego, CA, USA), anti- LAMP-2 (marker for lysosomes and late endosomes, Southern Biotechnology, Birmingham, AL, USA), anti-VAMP 2 (synaptobrevin/VAMP 2, marker for synaptic vesicles, Synaptic Systems, Göttingen, Germany), and anti-Rab3 (marker for synaptic vesicles, Synaptic Systems) antibodies were mouse monoclonal IgG. Secondary antibodies were Alexa Fluor 488-conjugated goat anti-rabbit IgG and Alexa Fluor 546-conjugated goat anti-mouse IgG (both from Molecular Probes, Eugene, OR, USA).

The anti-Aβ_42_ antibodies (Chemicon) are specific for C-termini of Aβ peptide, and they do not cross-react with full-length APP, APP C-terminal fragments (CTF), or with Aβ_40_[40]. We have also tested the specificity of anti-Aβ_40_ and anti-Aβ_42_ antibodies doing double immunostaining for Aβ and APP in control neuroblastoma cells. The anti-APP antibodies (Zymed, mouse anti-APP, clone LN27) recognize epitope within the first 200 amino acids in the APP N-terminus and react with all three known APP proteins. There is no cross-reactivity between Aβ and APP.

### Immunofluorescence microscopy

For immunofluorescence microscopy, cells on coverslips were washed twice in phosphate-buffered saline (PBS) and fixed in 4% neutral phosphate-buffered formaldehyde for 20 min at room temperature, rinsed in PBS, permeabilized with 0.1% saponin in PBS containing 5% serum for 20 min and incubated with primary antibodies for either single or double immunofluorescence for 1 h, followed by rinsing in PBS and 1 h incubation with secondary antibodies. Dilutions were 1:100 and 1: 400 for primary and secondary antibodies, respectively. For double immunostaining, different primary or secondary antibodies were applied simultaneously. The experiments were repeated at least three times.

After washing in PBS and distilled water, the specimens were mounted in Vectashield containing DAPI (Vector Laboratories, H-1200) and inspected with an inverted confocal laser scanning microscope (LSM 510 META, Zeiss) using a 488 nm argon laser and 543 nm helium-neon laser. For colocalization assessment, optical sections were no thicker than 0.6 μM. We also performed Nikon Microphot-SA fluorescence microscopy using a standard FITC / Texas Red double band-pass filter. Images were taken with a Hamamatsu ORCA 100 color digital camera (Hamamatsu, Japan). Images were prepared with Adobe Photoshop 7.0 (Adobe System).

## Results

**Figure 1:**
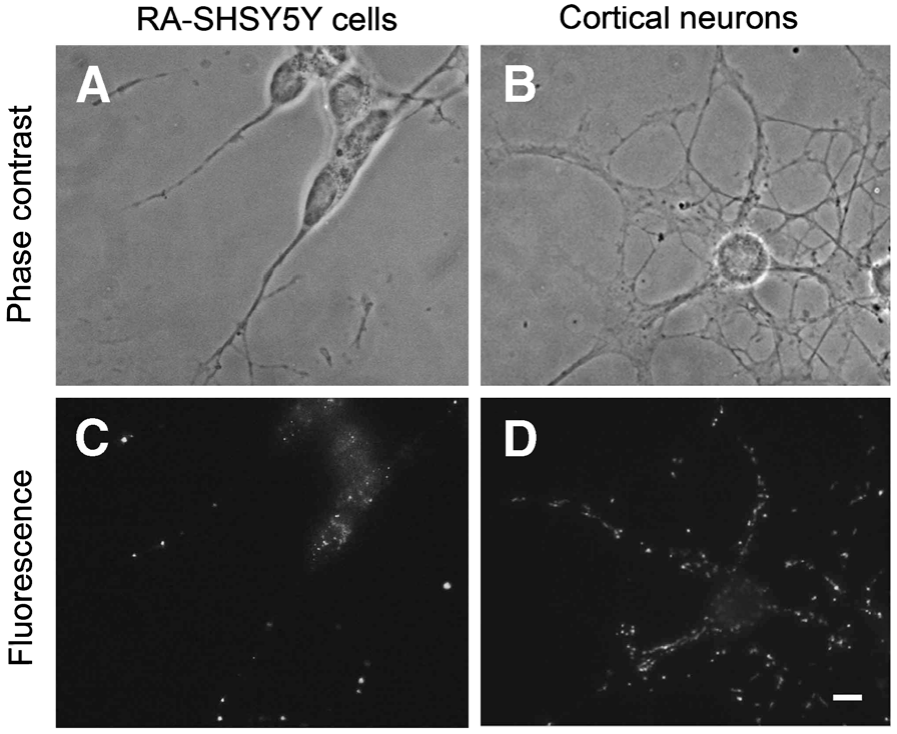
**Aβ**_**42**_** immunoreactivity in retinoic acid differentiated neuroblastoma cells and neonatal rat cortical neurons.** Phase contrast and fluorescence (greyscale) images of RA differentiated SH-SY5Y human neuroblastoma cells (**A**, **C**) and cultured rat cortical neurons (**B**, **D**), respectively. Aβ_42_ positive granules are bigger and more abundant in neurites than in perikarya. Bar, 50 nM.

Exposure of neuroblastoma SH-SY5Y cells to RA for two weeks resulted in their differentiation, which was characterized by the suppression of mitotic activity and development neurites (Figure 1A and C). Neonatal cortical neurons showed multiple anastomosing neurites. (Figure 1B and D). Aβ_42_ immunostaining showed intracellular localization of Aβ_42_ in both cell types. Aβ granules were larger and more abundant in neurites than in perikarya (Figure 1).

**Figure 2:**
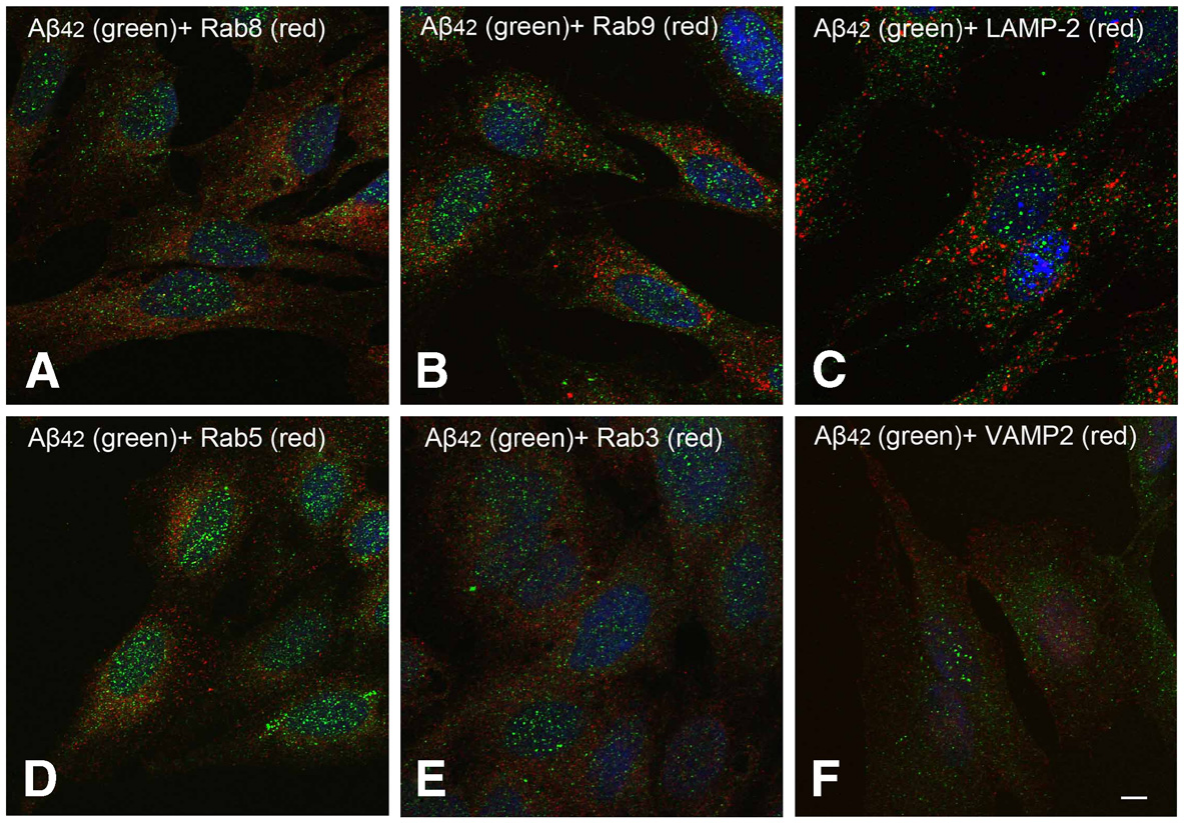
**Double immunostaining for Aβ**_**42**_** (green fluorescence) and different subcellular markers (red fluorescence) in RA differenatiated SH-SY5Y cells.** (**A**) Rab8 (Golgi derived vesicles marker), (**B**) Rab9 (trans-Golgi network, Golgi-derived vesicles and late endosome marker), (**C**) LAMP-2 (late endosome and lysosome marker), (**D**) Rab5 (early endosome marker), (**E**) Rab3 (exocytotic vesicle marker) and (**F**) VAMP2 (synaptobrevin, marker for synaptic vesicles) were used as subcellular organelle markers. Scale bar, 5 μm.

**Figure 3:**
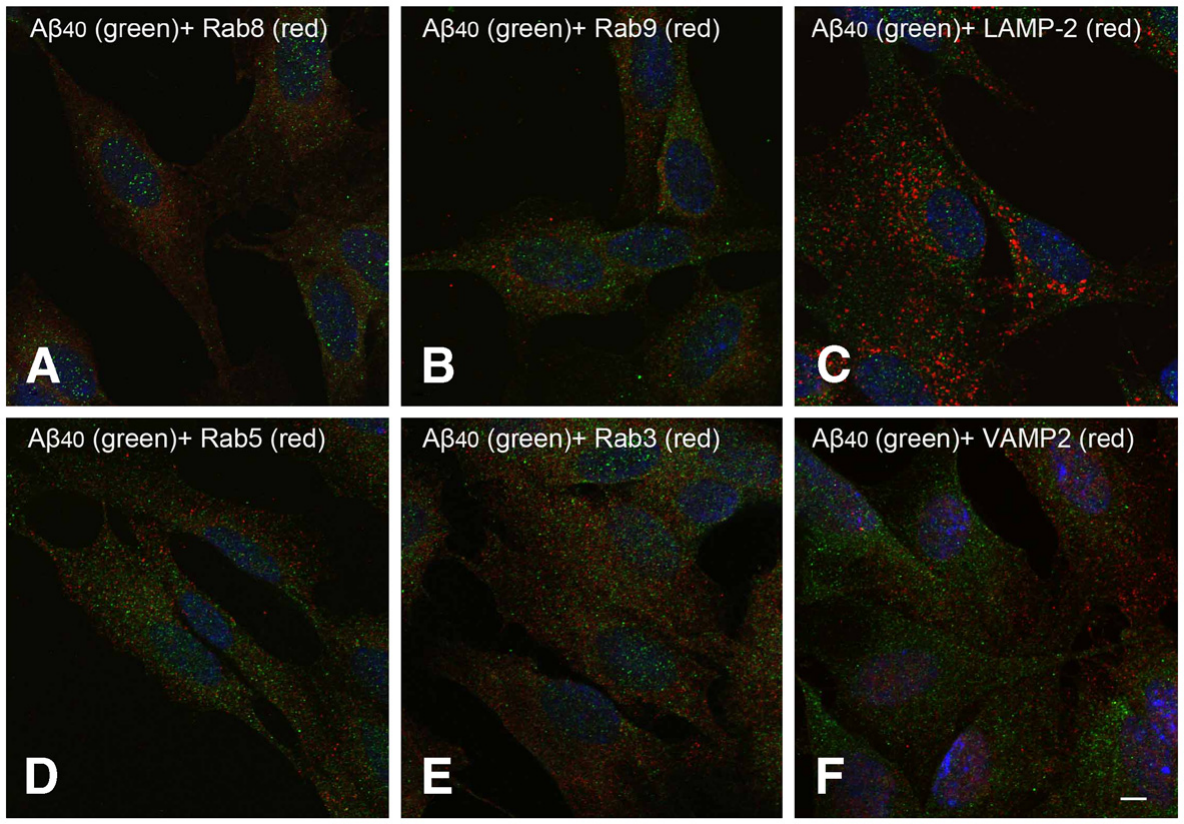
**Double immunostaining for Aβ**_**40**_** (green fluorescence) and different subcellular markers (red fluorescence) in RA differenatiated SH-SY5Y cells.** (**A**-**F**), see Figure 2. Scale bar, 5 μm.

To investigate intracellular localization of Aβ and its relationship with the lysosomal system and other organelles, RA differentiated neuroblastoma cells cultured under normal conditions were double immunostained for monomeric Aβ (Aβ_40_ and Aβ_42_) and different organelle-specific proteins. As shown in Figure 2, very few Aβ_42_ positive granules is colocalized with rab8 (TGN and Golgi derived vesicles marker), rab9 (TGN and late endosome marker), LAMP-2 (late endosome and lysosome marker), rab5 (early endosome marker), rab3 (exocytotic vesicle marker) or VAMP2 (or synaptobrevin, marker for synaptic vesicles). The overwhelming majority of Aβ_42_ granules were not colocalized with any of the markers. As shown in Figure 3, the intracellular distribution of Aβ_40_ is more diffused than that of Aβ_42_. Double immunostaining of Aβ_40_ and different organelle markers showed similar results regarding colocalization with organelles (Figure 3).

To study Aβ localization in relation with different subcellular compartments, we performed immunogold electron microscopy using antibodies for Aβ_40_ and Aβ_42_. Low amount of Aβ labeling was found in the endoplasmic reticulum, Golgi complexes, lysosomal compartment and also mitochondria, but it was particularly abundant in the cytosol, usually in the form of clusters (Zheng et al., unpublished results).

**Figure 4:**
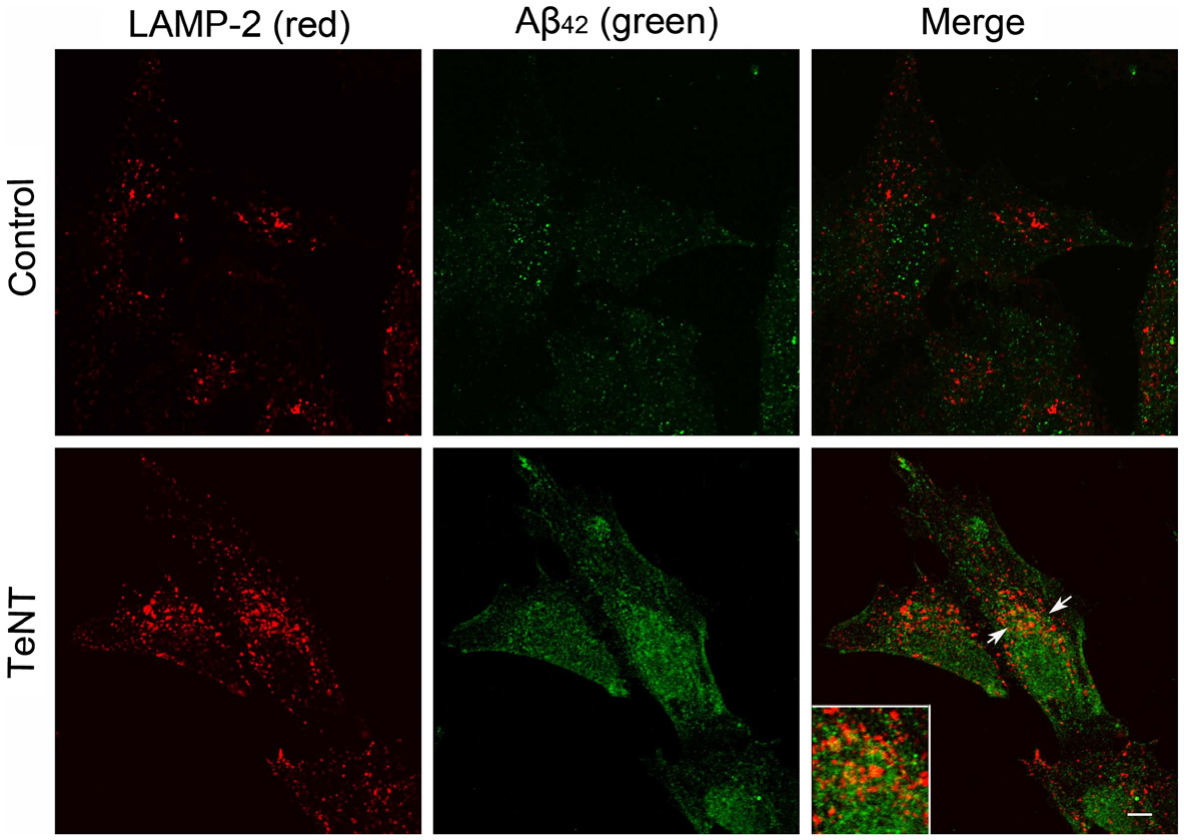
**Double immunostaining for Aβ**_**42**_** (green fluorescence) and lysosomal/late endosomal marker LAMP2 (red fluorescence) in RA differenatiated SH-SY5Y cells cultured under normal conditions or exposed to exocytosis inhibitor tetanus toxin (TeNT, 5 nM) for 24 h*.*** Both Aβ_42_ and LAMP-2 specific fluorescence are increased in tetanus toxin exposed cells, and the colocalization of Aβ_42_ with LAMP-2 positive structures (arrow and corresponding inset) is increased after TeNT treatment. Scale bar, 5 μm.

To study whether Aβ relation to lysosomes depends on its secretion, RA differentiated neuroblastoma cells were exposed to the exocytosis inhibitor TeNT, followed by double immunostaining for Aβ_42_ and LAMP-2. Cells were cultured under normal conditions (control) or treated with 5 nM TeNT for 24 h. The staining for both Aβ_42_ and LAMP-2 was brighter after the treatment with TeNT, suggesting the increase in the amount of intracellular Aβ_42_ as well as in the size of the lysosomal compartment. Both the size and the number of Aβ_42_-positive granules was increased after TeNT administration. Furthermore, although most Aβ_42_ granules were still found extralysosomally, more of them than in untreated cells were colocalized with LAMP-2 positive structures (Figure 4).

**Figure 5:**
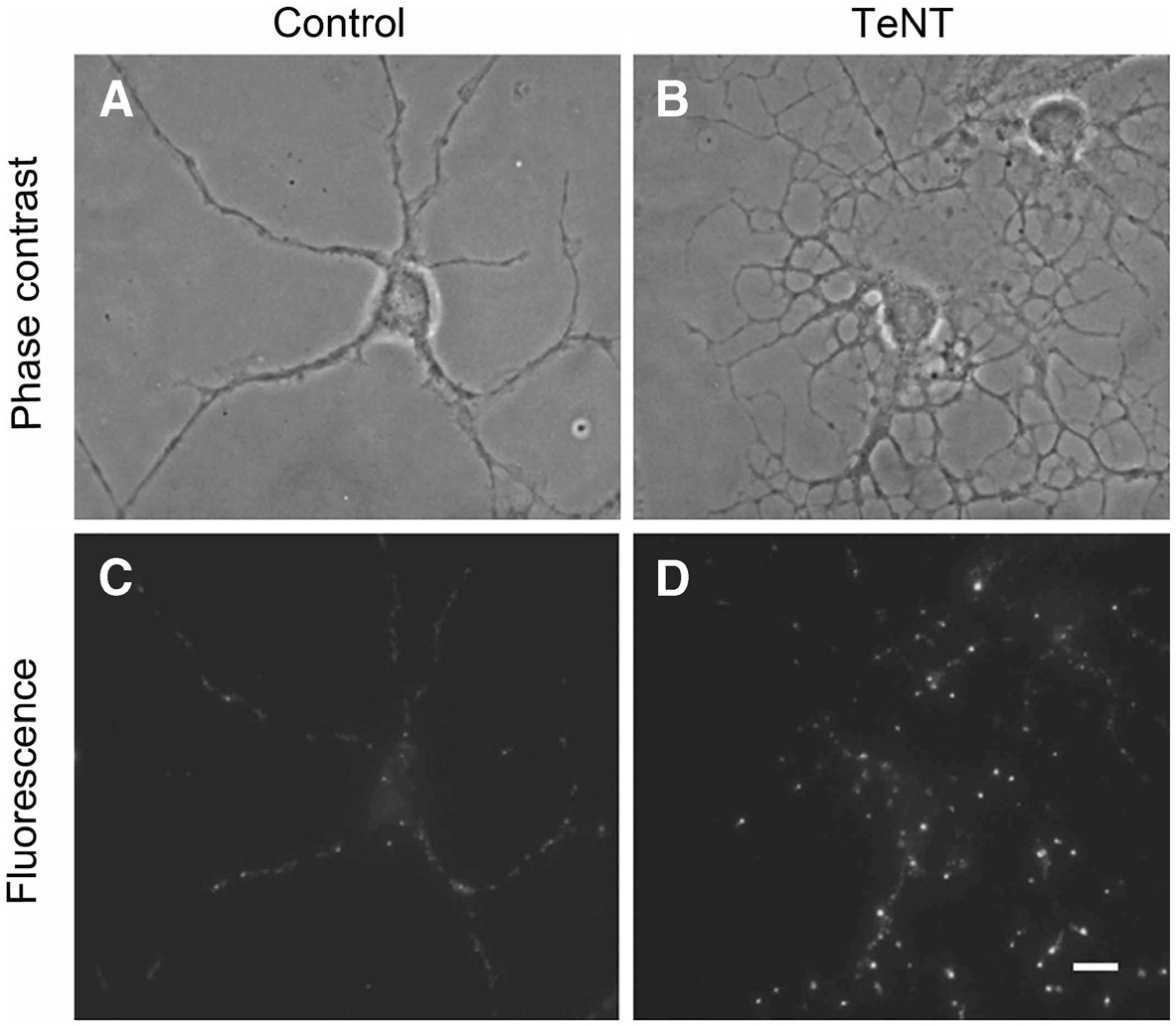
**Aβ**_**42**_** immunoreactivity (greyscale) of neonatal rat cortical neurons (A, C) cultured (under normal conditions (control) or (B, D) exposed to 20 nM exocytosis inhibitor tetanus toxin (TeNT) for 24 h.** (**A** and **B**) phase contrast images, (**B** and **D**) fluorescence images of corresponding cells. Both drugs induce intracellular accumulation of Aβ_42_. Bar, 50 nM. Intraneuronal Aβ_42_ is increased and accumulated in the neurites.

The effect of exocytosis inhibition on the intracellular distribution of Aβ_42_ was also studied using neonatal cortical neurons, which were exposed to 20 nM TeNT for 24 h. Phase contrast images show increased neuronal damage after TeNT treatment as compared to controls, while immunofluorescence microscopy reveals larger and more abundant Aβ_42_ positive granules along the neuritis, reflecting disturbed Aβ secretion and intraneuronal Aβ accumulation (Figure 5).

## Discussion

A large number of studies have explored the intracellular sites of Aβ production, mostly in AD models. Aβ_42_ and Aβ_40_ monomers have been previously demonstrated in ER [6-8], TGN [41] and post-TGN secretory vesicles [8], mitochondria [42], endosomes [27], lysosomes [43], multivesicular bodies (MVB) [44], and cytosol [12,45-47]. However, little is known about intracellular localization of Aβ in normal conditions, when Aβ is not overproduced.

In this study, we demonstrated that in differentiated neuroblastoma cells cultured under normal in vitro conditions, only little Aβ (including Aβ_42_ and Aβ_40_) showed colocalization with organelles such as TGN, Golgi-derived vesicles, early and late endosomes, lysosomes, or exocytotic vesicles, while the greater part of Aβ was located in the cytosol or in undetermined compartments.

The absence of major Aβ immunoreactivity in these cellular compartments, in which it was found in AD, as well as in cellular and in vivo AD models, suggests that, under normal conditions, this peptide is either relocated, or degraded, or secreted extracellularly. The fact that lysosomes showed little Aβ immunoreactivity would suggest that cells are able to perform a rapid proteolytic digestion of this peptide under normal biological conditions. In support of this hypothesis, we have previously shown that inhibition of lysosomal enzymes induces Aβ accumulation within the lysosomal compartment [29].

In addition, we have found that inhibition of exocytosis by TeNT induced a general increase of intracellular Aβ, both intra- and extralysosomal. As we previously reported [30], the intralysosomal Aβ accumulation can be mediated by enhanced Aβ autophagy. It is also possible that inhibition of exocytosis results in Aβ accumulation along the secretory pathway, including ER, Golgi apparatus, transport visicles and secretory vesicles [48].

Although under normal conditions late endosomes and lysosomes seem to be free of Aβ, this is not the case for AD neurons, in which Aβ has been demonstrated intralysosomally [10,27,28]. It is not clear what causes these changes and how Aβ relocation to lysosomes contributes to the pathogenesis of AD. One possible explanation is that oxidative stress might enhance autophagy, leading to intralysosomal Aβ accumulation, consequent lysosomal membrane damage and release of lysosomal enzymes to the cytosol, culminating in apoptosis [29,30].

In AD, Aβ has been shown to accumulate within lysosomes, apparently promoting neuronal death through lysosomal destabilization [22,25,49]. As we previously demonstrated, intralysosomal Aβ accumulation can be triggered by oxidative stress and consequent activation of macroautophagy [29,30]. On the other hand, Aβ has been shown to induce oxidant-mediated autophagic cell death in cultured cells [50], while antioxidants can protect cells from Aβ-mediated oxidative damage [51].

The fact that in the majority of AD cases there is no consistent overproduction of Aβ suggests that deficits in its degradation could lie behind the pathogenesis of the disease. On the other hand, intracellular accumulation of Aβ is proposed to compromise normal neuronal function in AD. Our findings demonstrate that, under normal conditions, intracellular Aβ (including Aβ_42_ and Aβ_40_) is mainly associated with cytosolic structures and, to a large extent, is secreted from the cells. They may also suggest that deficits in secretion or lysosomal processing would result in intracellular Aβ accumulation and its translocation to the cellular organelles, as seen in AD and its models [12,21,52,53]. Our finding may contribute to better understanding of AD pathogenesis, and may help develop new therapeutic strategies against AD (reviewed in [54]).

## Abbreviations

AD: Alzheimer disease; Aβ: Amyloid β-protein; APP: Amyloid Precursor Protein; ER: Endoplasmic Reticulum; PBS: Phosphate-buffered Saline; LAMP-2: Lysosomal Associated Membrane Protein-2; MPRs: Mannose 6-phosphate Receptors; RA: Retinoic Acid; TeNT: Tetanus Toxin; TGN: Trans Golgi network.

## Competing interests

The author(s) declare that they have no competing interests.

## Authors’ contributions

LZ carried out experiments, participated in manuscript writing and revision, AC-M participated in project design and manuscript writing, MH participated in project design, FJ participated in part of experiments, JM participated in project design, AT participated in project design, manuscript writing and revision. All authors read and approved the final manuscript.
